# Prognostic Implications of Left Ventricular Ejection Fraction and Left Ventricular End-Diastolic Diameter on Clinical Outcomes in Patients with ICD

**DOI:** 10.3390/jcdd9120421

**Published:** 2022-11-28

**Authors:** Sijing Cheng, Yu Deng, Hao Huang, Xi Liu, Yu Yu, Xuhua Chen, Min Gu, Hongxia Niu, Wei Hua

**Affiliations:** State Key Laboratory of Cardiovascular Disease, Arrhythmia Center, Fuwai Hospital, Chinese Academy of Medical Sciences and Peking Union Medical College, No.167 North Lishi Road, Beijing 100037, China

**Keywords:** implantable cardioverter defibrillator, left ventricular ejection fraction, left ventricular end-diastolic diameter, clinical outcomes

## Abstract

Background: Left ventricular ejection fraction (LVEF) is a suboptimal indicator of risk stratification for patients with an implantable cardioverter defibrillator (ICD). Studies have shown that left ventricular end-diastolic diameter (LVEDD) was associated with all-cause mortality and ventricular arrhythmias. We examined the quantified prognostic value of LVEF and LVEDD for clinical outcomes, respectively. Method: This study retrospectively enrolled patients with ICD implantation in a single center. The associations between LVEF or LVEDD and all-cause mortality and appropriate shocks were analyzed using Cox regression and Fine-gray competing risk regression, respectively. Result: During a median follow up of 59.6 months, 168/630 (26.7%) patients died. LVEF and LVEDD were strongly associated with all-cause mortality (LVEF per 10%: HR 0.77, 95%CI 0.64–0.93, *p* = 0.006; LVEDD per 10 mm: HR 1.54, 95%CI 1.27–1.85, *p* < 0.001). After a median interrogation time of 37.1 months, 156 (24.8%) patients received at least one shock. LVEF was not associated with appropriate shock, whereas larger LVEDD (per 10 mm) was significantly associated with a higher risk of shock (HR: 1.27, 95%CI 1.06–1.52, *p* = 0.008). The addition of LVEF or LVEDD to clinical factors provided incremental prognostic value and discrimination improvement for all-cause mortality, while only the addition of LVEDD to clinical factors improved prognostic value for shock intervention. Conclusions: Baseline LVEF and LVEDD show a linear relationship with all-cause mortality in patients with ICD. However, whereas LVEF is not associated with shock, a linear relationship exists between LVEDD and appropriate shock. LVEDD adds more predictive value in relation to all-cause mortality and appropriate shocks than LVEF.

## 1. Introduction

Sudden cardiac death (SCD) accounts for approximately 25% of cardiovascular death, and implantable cardioverter defibrillator (ICD) effectively reduces mortality related to sudden death. Current ventricular arrhythmias (VA) and SCD guidelines recommend primary ICD implantation in symptomatic (New York Heart Association (NYHA) function class II-III) ischemic cardiomyopathy (ICM)/non-ischemic cardiomyopathy (NICM) patients and left ventricular ejection fraction (LVEF) ≤35% after ≥3 months of optimal medical treatment [1,2]. LVEF is the core indicator of risk stratification for patients with a risk of SCD, and the cut-off of 35% is derived from the pivotal clinical trials conducted approximately 20 years ago [3,4,5].

Other echocardiographic characteristics such as LV mass, left atrial dimension and left ventricular end-systolic dimension have been shown to be associated with clinical outcomes [6]. Among these, baseline left ventricular end-diastolic diameter (LVEDD) has been associated with all-cause mortality and SCD in the general population and heart failure patients [7,8,9,10,11]. However, the impact of LVEF and LVEDD on all-cause mortality and SCD has not been thoroughly investigated in patients with ICD.

Therefore, the aim of this study is to quantify the prognostic value of LVEF and LVEDD for clinical outcomes, respectively. We also investigated the incremental value of adding LVEF and LVEDD to the established clinical risk predictors.

## 2. Methods

### 2.1. Population

We retrospectively included patients from a maintained ICD database at Fuwai Hospital from March 2010 to September 2020. Eligible patients were age older than 18, had complete echocardiography examination and had a diagnosis of ICM or NICM. For the indication of ICD, primary prevention was defined as in symptomatic ICM/NICM patients with NYHA function class II-III and LVEF ≤ 35% following optimal medical treatment for at least 3 months. The secondary prevention was defined as prophylactic ICD implantation to prevent sudden cardiac death in patients with a history of life-threatening ventricular arrhythmia [1,2]. ICM was defined as significant stenosis (≥50% of left main or ≥70% of major epicardial coronary artery) on coronary angiography or with evidence of myocardial infarction. We excluded patients who were lost of follow-up, without current implantation indication of ICD [1], or who had undergone device replacement. This study was conducted in accordance with the Declaration of Helsinki and approved by the institutional review board and ethics committees. All patients provided written informed consent.

### 2.2. Echocardiography

Electrocardiogram-triggered two-dimensional echocardiographic examination was performed before the implantation of ICD by experienced sonographers using commercially available equipment (GE Vivid) and interpreted by experienced cardiologists. LVEF was calculated using the biplane summation-of-disks method (modified Simpson’s rule) in the apical four- and two- chamber views. LVEDD was measured at a level just below the mitral valve leaflet tips in the parasternal long-axis view [12].

### 2.3. Follow-Up and Outcomes

ICD patients underwent routine interrogation and follow up at Fuwai hospital. The primary endpoint of this study was all-cause mortality. The secondary endpoint was the occurrence of appropriate ICD shock as a substitute for SCD. Appropriate ICD shock was defined as shocks for ventricular tachycardia (VT) or ventricular fibrillation (VF). Because this study included patients with secondary prevention, the ICD programming parameters for VT and VF varied among patients.

### 2.4. Statistical Analysis

Continuous variables were presented as means and standard deviation (SD) or medians and interquartile range (IQR) as appropriate. Categorical variables were presented as counts and percentages. Differences among quintiles were analyzed using one-way ANOVA or Kruskal-Wallis test for continuous variables and Pearson chi-square test or Fisher’s exact test for categorical variables. For variables missing less than 5%, they were considered to be missing at random, and random forest imputation was used. The correlation between potential predictors was performed by calculating Pearson’s correlation coefficient. When predictors demonstrated a high degree of collinearity, only one factor was considered to be included in the regression model. Multivariable Cox regressions were used to investigate the relationship of LVEF and LVEDD to all-cause mortality. Fine-Gray analyses accounting for all-cause mortality as a competing risk were used to investigate their relationships to appropriate ICD shocks. We adjusted models for important clinical predictors as well as for covariates that were significant in univariable analysis. For the Cox regression analysis, our basic model (model 1) started with several clinical predictors based on prior research and univariable Cox regression results: age, sex, BMI, systolic blood pressure, NYHA class >II and indication of ICD. Model 2 was additionally adjusted for comorbidities, including complete right bundle branch block (RBBB), atrial fibrillation (AF), diabetes, and ICM. Model 3 was additionally adjusted for medications including digoxin, diuretic and ACEI/ARB/ARNI compared with model 2. Model 4 further included eGFR, albumin and NT-proBNP. For the analysis of the secondary endpoint, model 1 adjusted for covariates mentioned above in Cox regression model 1, as well as for smoking and alcohol. The additional variables in models 2– 4 were the same as in the Cox regression model.

To evaluate the association of LVEF and LVEDD with the endpoints, we first stratified the population into quintiles according to LVEF and LVEDD, respectively. Then P for linear trend was calculated. The potential non-linearity was tested using penalized spline terms and the association between the echocardiographic parameter and the risk of the endpoint was plotted using penalized splines. Secondly, we treated the echocardiographic parameter continuously if it did not violate the linearity assumption of the Cox regression.

The performance of models was compared using C-statistic improvement. The likelihood ratio test was used to evaluate the fit of the models. Category-free net reclassification index (NRI) and integrated discrimination index (IDI) were assessed to measure the prediction improvement of LVEF and LVEDD. Possible interactions of LVEF or LVEDD with sex, the indication of ICD, ICM, and CLBBB were tested. Furthermore, we performed sensitivity analyses examining the robustness of the results using complete-case analysis. Statistical analysis was performed in R version 4.1.2 (Vienna, Austria). Multivariable Cox regressions were performed using the survival (version 3.2-13) package and competing risk models were fitted using the cmprsk (version 2.2-10) package.

## 3. Results

### 3.1. Baseline Characteristics

**Figure 1:**
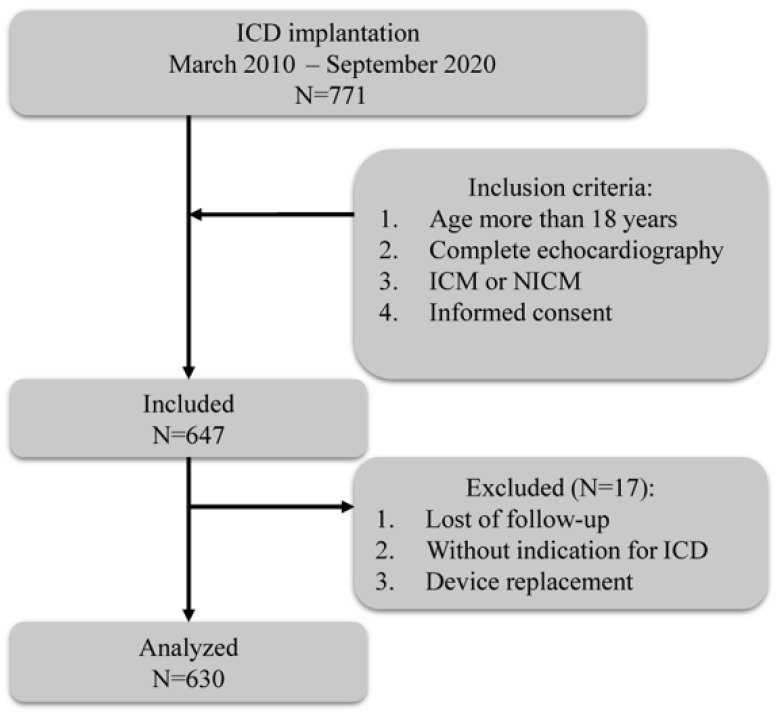
Flowchart of the study. ICD, implantable cardioverter defibrillation; ICM, ischemic cardiomyopathy; NICM, non-ischemic cardiomyopathy.

Among 771 patients, 647 fulfilled the inclusion criteria. Of these, 17 patients were lost to follow-up, without indication of ICD or having undergone device replacement, and 630 patients were finally included for analysis (Figure 1).

The baseline characteristics of the population are summarized in Table 1. The mean age was 60.7 years, 83.7% were men, 54.4% had a diagnosis of ICM, and 23.0% were primary prevention of SCD. The mean LVEF and LVEDD was 37.3% and 64.2 mm, respectively. Patients were stratified into five groups according to LVEF: 126 patients were in Q1 (LVEF < 27%), 122 patients were in Q2 (27–33%), 125 patients were in Q3 (33–38%), 128 patients were in Q4 (38–48%) and 129 patients were in Q5 (LVEF ≥ 48%). Notable differences between the LVEF quintiles were seen in age, systolic blood pressure, NYHA functional class >II, hypertension, AF, ICM, hyperlipidemia, primary indication, use of digoxin, diuretic, amiodarone, eGFR and NT-proBNP (Table S1). LVEDD was also categorized into Q1 (<57 mm, N = 121), Q2 (57–61 mm, N = 109), Q3 (61–66 mm, N = 141), Q4 (66–72 mm, N = 131), and Q5 (≥72 mm, N = 128). The differences between LVEDD quintiles were also included in Table S1. Larger LVEDD was associated with younger age, male sex, lower systolic blood pressure, worse NYHA functional class, less comorbidities of ICM and hyperlipidemia, higher prevalence of complete left bundle branch block (CLBBB) and primary prevention of SCD. We found an excellent reverse correlation between LVEF and LVEDD in this population, with Pearson correlation coefficient = −0.62 (*p* < 0.001).

### 3.2. Follow-Up

**Figure 2:**
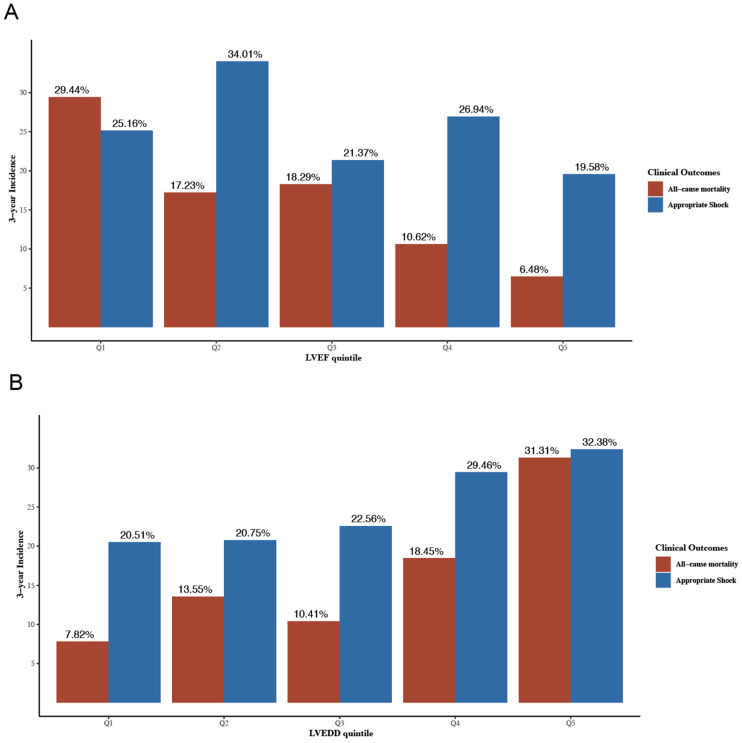
3-year cumulative incidence of all-cause mortality and appropriate shock according to LVEF quintiles (**A**) and LVEDD quintiles (**B**). LVEDD, left ventricular end-diastolic diameter; LVEF, left ventricular ejection fraction. LVEDD, left ventricular end-diastolic diameter; LVEF, left ventricular ejection fraction.

During a median follow-up time of 59.6 months (interquartile range 55.3–63.7), 168 (26.7%) patients reached the primary endpoint. A total of 156 (24.8%) patients received at least one appropriate shock during a median pacemaker interrogation time of 37.1 months (interquartile range 37.1–42.9). Results of univariable cox regression for all-cause mortality and appropriate shock are shown in Table S2. Figure 2 shows the 3-year cumulative incidences of all-cause mortality and appropriate shock according to LVEF (Figure 2A) and LVEDD (Figure 2B) quintiles. The multivariable regression results according to echo parameters quintiles and clinical outcomes in model 4 are shown in Table S3.

### 3.3. Association of LVEF with Mortality

**Figure 3:**
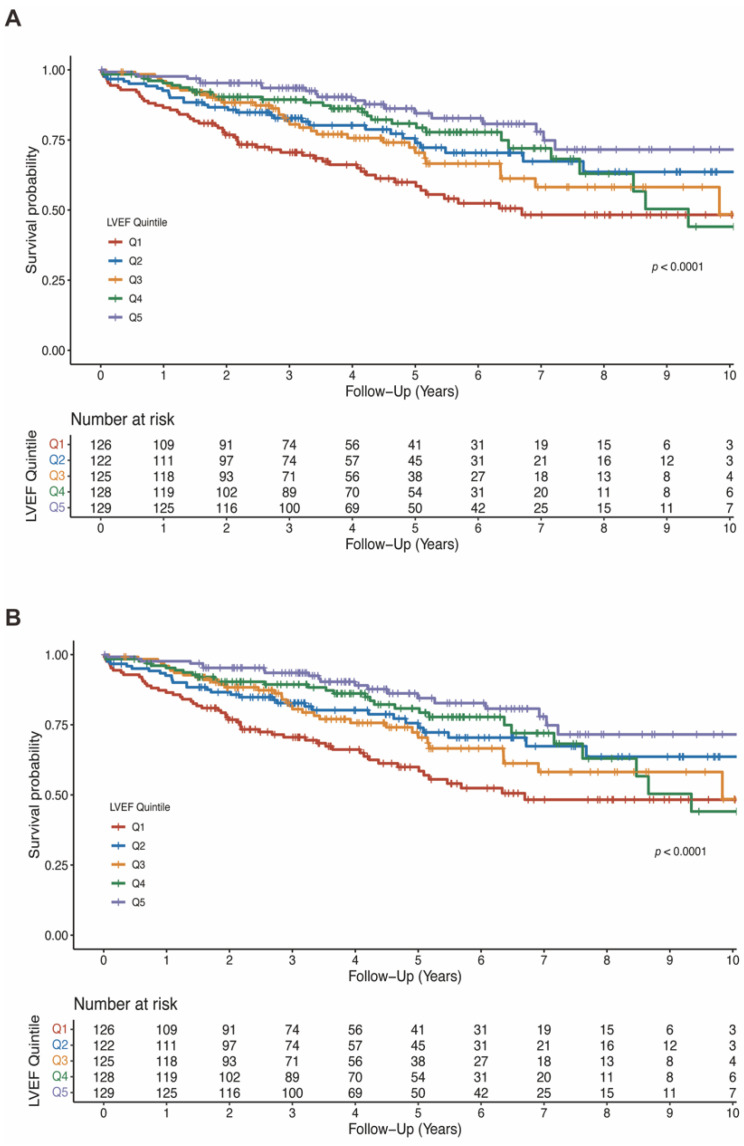
Kaplan-Meier estimates of survival according to LVEF quintiles (**A**) and LVEDD quintiles (**B**). LVEDD, left ventricular end-diastolic diameter; LVEF, left ventricular ejection fraction.

The mean LVEF in the entire population was 37.3 ± 11.7%. LVEF was significantly lower in patients who died than patients who survived (33.7 ± 10.2% vs. 38.6 ± 11.9%, *p* < 0.001; Table S4). As shown in Figure 3A, a lower baseline LVEF quintile was significantly associated with all-cause death (log-rank test *p* < 0.0001). In the model 4 adjusting for baseline characteristics, comorbidities, medication and biomarkers, higher LVEF was associated with lower risk of all-cause mortality when compared with Q1 (HR 0.64, 95%CI 0.41–1.01, *p* = 0.056 for Q2; HR 0.72, 95%CI 0.45–1.15, *p* = 0.165 for Q3; HR 0.64, 95%CI 0.38–1.07, *p* = 0.086 for Q4; HR 0.49, 95%CI 0.26–0.92, *p* = 0.027 for Q5). The summarized results of multivariable cox analyses for LVEF in all four models are shown in Table 2.

**Figure 4:**
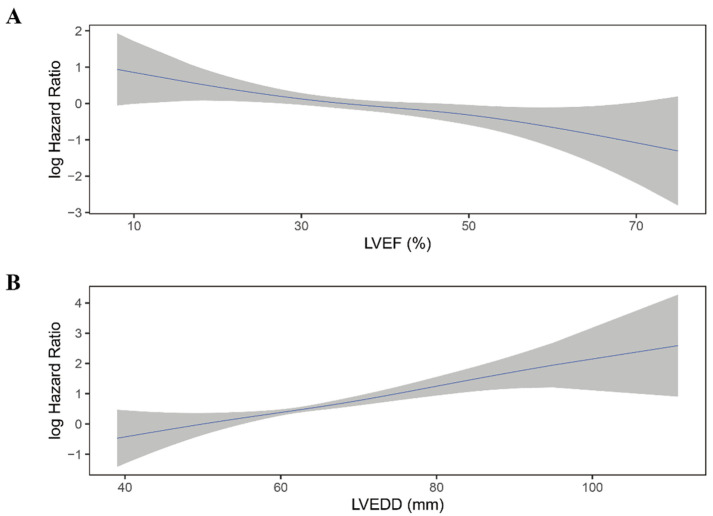
Continuous log HR plot for the relationship between echocardiographic parameters and all-cause mortality ((**A**) LVEF; (**B**) LVEDD). Blue line: log HR; grey area: 95%CI. LVEDD, left ventricular end-diastolic diameter; LVEF, left ventricular ejection fraction.

Moreover, LVEF quintiles showed a clear linear trend for all-cause mortality in all four models (P for trend < 0.05). The association between LVEF as a continuous variable and the risk of all-cause mortality was plotted (Figure 4A). As LVEF did not violate the linearity assumption of the Cox regression model (P for non-linearity > 0.05), we then included LVEF as a continuous variable in model 1 to model 4. LVEF was an independent predictor for all-cause mortality in Cox regression analysis and consistent in all models (HR for per 10% increase in LVEF: model 1: 0.76; 95% 0.64–0.89; *p* = 0.001; model 2: 0.76; 95%CI 0.64–0.90; *p* = 0.002; model 3: 0.75; 95%CI 0.62–0.89; *p* = 0.001; model 4: 0.77; 95%CI 0.64–0.93; *p* = 0.006) (Table 2).

### 3.4. Association of LVEDD with Mortality

The mean LVEDD was 64.2 ± 9.7 mm and was significantly larger in patients who died than survived (67.3 ± 10.0 mm vs. 63.1 ± 9.4 mm, *p* < 0.001; Table S4). Similarly, lower LVEDD quintiles were associated with lower risk of all-cause mortality (log-rank test *p* < 0.001; Figure 3B). In fully adjusted model, LVEDD was independently associated with all-cause mortality (compared to Q5: HR 0.39, 95%CI 0.22–0.70, *p* = 0.002 for Q1; HR 0.61, 95%CI 0.36–1.02, *p* = 0.058 for Q2; HR 0.41, 95%CI 0.26–0.66, *p* < 0.001 for Q3; HR 0.54, 95%CI 0.35–0.85, *p* = 0.008 for Q4; Table 3).

Similar to LVEF, LVEDD quintiles showed a trend toward higher all-cause mortality risk (*p* < 0.001) and did not violate the linearity assumption of continuous variable (Figure 4B; P for non-linearity > 0.05). When considered as a continuous variable, Larger LVEDD was significantly associated with a higher risk of all-cause mortality (HR for per 10 mm increase in LVEDD: model 1: HR 1.54; 95%CI 1.30–1.82; *p* < 0.001; mode2: HR 1.56; 95%CI 1.31–1.87; *p* < 0.001; model 3: HR 1.57; 95%CI 1.31–1.88; *p* < 0.001; model 4: HR 1.54; 95%CI 1.27–1.85; *p* < 0.001) (Table 3).

### 3.5. Association of LVEF and LVEDD with Appropriate Shock

**Figure 5:**
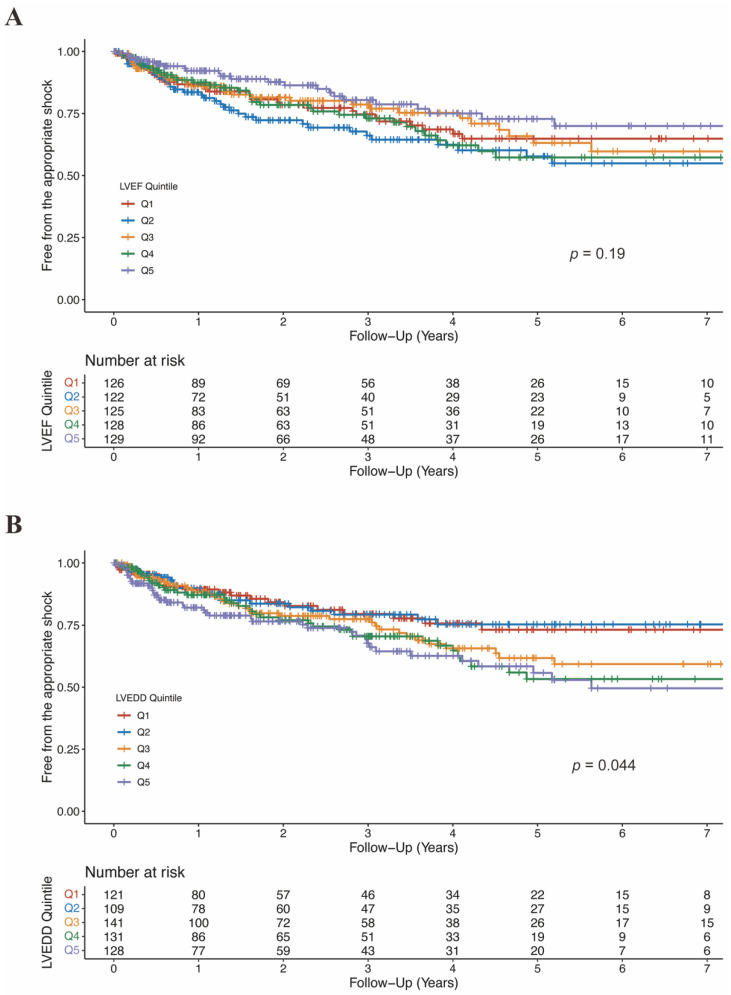
Free from the appropriate shock by LVEF quintiles (**A**) and LVEDD quintiles (**B**). CI, confidence interval; LVEDD, left ventricular end-diastolic diameter; LVEF, left ventricular ejection fraction.

**Figure 6:**
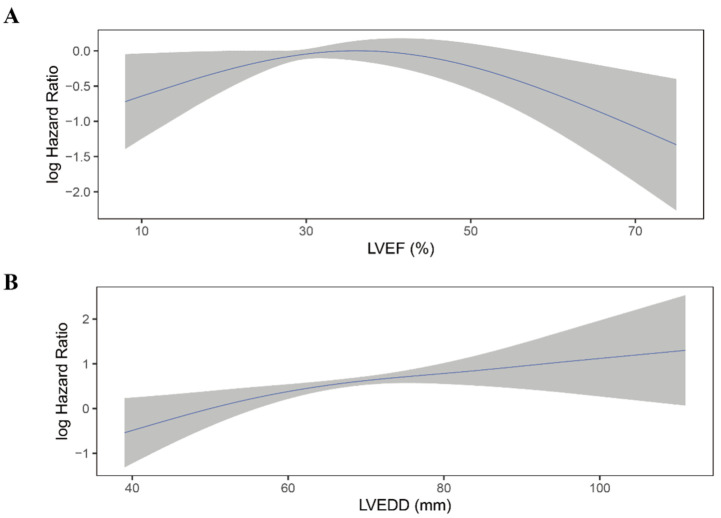
Continuous log HR plot for the relationship between echocardiographic parameters and appropriate shock ((**A**) LVEF; (**B**) LVEDD). Blue line: log HR; grey area: 95%CI. CI, confidence interval; LVEDD, left ventricular end-diastolic diameter; LVEF, left ventricular ejection fraction.

There was no significant difference in LVEF between patients who experienced shock and who not (36.1 ± 10.2% vs. 37.2 ± 12.1%, *p* = 0.12; Table S4). Moreover, when categorized into quintiles, LVEF was not associated with the risk of appropriate ICD shock (Figure 5A; Table 4). As shown in Figure 6A, the association between LVEF and the risk of appropriate shock violated the linearity assumption; therefore, we did not treat LVEF as a continuous variable.

LVEDD was significantly larger in patients who experienced appropriate shock than those who did not (66.1 ± 9.2 mm vs. 63.6 ± 9.8 mm, *p* = 0.004; Table S4). When categorized into quintiles, there was an increasing risk of appropriate shock as LVEDD increased (Figure 5B). In the fully adjusted model (model 4), LVEDD was an independent predictor of appropriate shocks (compared to Q5: HR 0.55, 95%CI 0.31–0.99, *p* = 0.048 for Q1; HR 0.47, 95%CI 0.26–0.85, *p* = 0.013 for Q2; HR 0.83, 95%CI 0.53–1.29, *p* = 0.405 for Q3; HR 0.78, 95%CI 0.50–1.20, *p* = 0.260 for Q4; Table 5). Then LVEDD was used as a continuous variable (P for trend = 0.014, P for non-linearity = 0.446; Figure 6B). Multivariable competing risk analyses demonstrated that the HR for the occurrence of appropriate shock became higher with increasing LVEDD in the fully adjusted model (LVEDD per 10 mm: HR 1.27; 95%CI 1.06–1.52; *p* = 0.008; Table 5).

### 3.6. Incremental Prognostic Value of LVEF and LVEDD in Multivariable Models

To compare the incremental prognostic value of LVEF and LVEDD, we first constructed the corresponding clinical models including all variates in the aforementioned models except for the echocardiographic parameter. For the primary endpoint of all-cause mortality, the addition of LVEF resulted in modest improvement in model fit (χ2 increased by 7.779; *p* = 0.005) and discrimination improvement (IDI 0.015, 95%CI 0.002–0.038, *p* = 0.007) in the fully adjusted model (model 4). The addition of LVEDD in model 4 resulted in a significant increase in χ2 by 19.574 (*p* < 0.0001) and IDI by 0.037 (95%CI 0.011–0.069, *p* < 0.0001). As for category-free reclassification analysis for all-cause mortality, the addition of LVEF and LVEDD to clinical variables resulted in a significant reclassification improvement of 12.9% (95%CI 0.7%–24.8%) and 24.3% (95%CI 9.1%–32%), respectively (Table S5).

For the secondary endpoint of appropriate shock, LVEDD rather than LVEF was an independent predictor of the shock. The addition of LVEF to a clinical model did not provide incremental prognostic value or discrimination improvement. However, the addition of LVEDD to the fully adjusted model significantly increased the model χ2 (5.814; *p* = 0.02) and showed a trend towards improved discrimination (IDI 0.013, 95%CI-0.006–0.044, *p* = 0.140). For NRI, the addition of LVEF and LVEDD resulted in nonsignificant reclassification improvement of 10.2% (95%CI-5.3%–18.5%) and 13.6% (95%CI-0.6%–20.2%) (Table S5).

### 3.7. Subgroup and Sensitivity Analyses

There were no significant interactions between LVEF/LVEDD and sex, the indication of ICD and cardiomyopathy etiology for the risk of mortality and appropriate shock (Table S6). Analyses of sensitivity using complete-case were included in Table S7. Effect sizes in the fully adjusted model were similar to the entire population. LVEF was predictive for all-cause mortality (HR for per 10%: 0.78, 95%CI 0.63–0.96, *p* = 0.020) but not appropriate shock (P for trend: 0.230), while LVEDD was predictive for both all-cause mortality (HR for per 10 mm: 1.51, 95%CI 1.23–1.86, *p* < 0.001) and appropriate shock (HR for per 10 mm: 1.25, 95%CI 1.04–1.51, *p* = 0.017).

## 4. Discussion

The main findings of our study including patients with ICD implantation are as follows: (1) patients with lower LVEF or larger LVEDD had a higher risk of all-cause mortality during follow-up. (2) patients with larger LVEDD at baseline had a higher risk of appropriate shock. (3) Moreover, the addition of LVEDD to clinical models provided incremental prognostic information over LVEF for both endpoints. These findings indicate that LVEDD could be a helpful predictor of all-cause mortality and appropriate shock better than LVEF in patients with ICD implantation.

According to current guidelines, LVEF is the core indicator of the selection criterion for primary prevention [2]. LVEF was a strong, independent predictor of SCD and cardiovascular mortality in pivotal trials [5,13]. For HFrEF patients, ICD was associated with 23% relative risk reduction in all-cause mortality in pivotal RCTs [14]. However, the DANISH trial in which the inclusion criteria required LVEF ≤ 35%, failed to show the survival benefit of ICD in NICM patients [15]. Prior studies have shown that LVEF is neither highly sensitive nor specific in regard to SCD prediction [16,17]. New predictors for risk prediction of clinical outcomes are urgently warranted. A suitable marker for patients should therefore predict not only all-cause mortality but also the risk of arrhythmic risk. Our study shows that LVEDD could be a valuable marker to predict both clinical outcomes. Moreover, LVEF cutoff of 35% bypassed the rigorous steps of evaluation to test whether it is sufficient to predict clinical outcomes [17]. Our results provide the first detailed, quantified relationship between echocardiographic parameters (LVEF and LVEDD) and clinical outcomes. Our results suggest a linear relationship between LVEF and LVEDD to all-cause mortality; however, for the appropriate shocks, a linear association was only identified with LVEDD.

Several observational studies demonstrated that LVEDD was associated with the risk of all- cause mortality and ventricular arrhythmias in patients with ICD [7,18,19]. Sataka et al. showed that LVEDD ≥ 65 mm was an independent predictor of fatal arrhythmic events in patients with primary prevention for class I and class IIa indication in Japan [20]. Another study including 853 patients with only Biotronik ICD demonstrated that moderate LVEF dysfunction (35–45%) and left ventricular enlargement (LVEDD ≥ 60 mm) were associated with a higher risk of VA and cardiac death [21]. Moreover, in a sub-study of the Genetic Risk of Assessment of Defibrillator Events (GRADE) study, LVEDD was associated with appropriate shocks (HR for per 10 mm: 1.33, *p* < 0.001) and the composite endpoint of death, transplant and left ventricular assist device (HR for per 10 mm 1.40 *p* < 0.001) [7]. Our study supports these results. However, LVEF was found to be both associated with shocks (HR for per 1% 0.95, *p* = 0.0002) and the composite endpoint (HR for per 1% 0.95, *p* < 0.001) in this study [7]. This is different from our results. Of note, there were several key differences between the two studies. First, this study ignored the competing risk of death. Therefore, this could overestimate the risk of appropriate shocks and might distort the results [22]. Second, the differences in patients’ characteristics might contribute to the controversial results. The inclusion criterion was LVEF < 30%, and the majority of patients (74.61%) had the primary indication in the sub-study of the GRADE study. However, in our study, the mean LVEF was 37.3 ± 11.7% and 23.1% of patients had the primary indication. Subgroup analyses in our study indicated that there was no interaction between the predictive value of echocardiographic parameters and the indication of ICD.

Larger LVEDD represents dilation of the left ventricular. Left ventricular dilation reflects the alternations in the expression of connexins and stretch-mediated changes in ion channel function and adverse left ventricular remodeling, and could create a favorable milieu for re-entrant ventricular arrhythmias [23,24]. In animal models of dyssynchronous heart failure, LV end- diastolic volume was significantly larger in the heart failure (HF) model. Moreover, remodeling of K^+^ current and Ca^2+^ current and prolongation of action potential duration (APD) were shown in the dyssynchronous HF model, which might be cellular triggers and substrate for arrhythmias [23,25]. Another mechanism underlying these might be attributable to myocardial fibrosis. Prior study has demonstrated that NICM patients with fibrosis had higher left ventricular end-diastolic volumes and the myocardial fibrosis was independently associated with all-cause mortality and SCD [26,27].

In this study, calculation of LVEF and measurement of LVEDD were according to current recommendations [12]. However, the accuracy of LVEF calculation was in excess of ±10% for Simpson’s rule with echocardiography [28]. Thus, LVEF might have limited reproducibility, especially in 2D imaging, which is commonly used in clinical practice. In contrast, LVEDD was measured using an electronic caliper and a line at the certain level just below the mitral valve leaflet tips, which enhanced the reproducibility of the results.

For the optimal cut-off of LVEDD, previous studies applied different cut-off values according to the characteristics of patients. In CHART-1 study, LVEDD > 60 mm was associated with SCD in heart failure patients, while in CHART-2 study, LVEDD > 65 mm rather than 60 mm was a significant risk factor [8,20]. In our study, we decided not to define the optimal cut-off using the Youden index in receiver-operating characteristic, as this could lead to a type-I error inflation. Moreover, LVEDD represents a continuum of risk, and the aim of this study is to qualify the relationship of LVEDD and clinical outcomes. Thus, we moved from a dichotomous to a continuous risk stratification.

LVEDD and LVEF in this study were measured at baseline. Prior study has proven that 24% of NICM patients with prophylactic ICD implantation had left ventricular function improvement (LVEF increase ≥5% and LVEDD decrease ≥5 mm) within 1 year [29]. However, our study shows that LVEDD was still associated with clinical outcomes for almost ten years of follow-up, and future studies are warranted to assess the value of dynamic examination of LVEDD.

### Limitations

Our study has several limitations. First, the main limitation of the present study is its retrospective observation design. However, it reflects the real-world clinical practice and prospective research is needed in the future. Second, in our study, the appropriate shock was the secondary outcome as the substitute for SCD. We acknowledge that SCD is a more meaningful endpoint. Moreover, a study showed that ICD therapies are more frequent than sudden death, with approximately twice as many shocks as fatal arrhythmias [30]. However, we did not obtain the causes of death, thus we could only use appropriate shock as a surrogate for SCD. Third, echocardiography was measured at baseline, and the association of temporal change of LVEDD and clinical outcomes was not evaluated. In our study, LVEDD was still associated with clinical outcomes after 59.6 months of follow-up. Future studies are needed to assess the relationship between LVEDD temporal change and clinical outcomes. Fourth, we included a mixed population of patients with ischemic and non- ischemic cardiomyopathy and primary and secondary prevention indication, while there were no significant interactions in subgroup analysis. Fifth, we did not have the genetic testing results to perform a subgroup analysis. Finally, as granular CMR data were not available in this study, no further relationship between LVEDD and fibrosis could be investigated.

## 5. Conclusions

In conclusion, in patients with ICD implantation, both LVEDD and LVEF showed a linear relationship with all-cause mortality. However, LVEF was not associated with appropriate shock. In contrast, LVEDD still showed a linear relationship with appropriate shock. Moreover, LVEDD had a strong incremental prognostic value for outcomes over clinical factors. Thus, further studies are needed to validate the results, and LVEDD may be incorporated into the risk stratification of patients with ICD implantation.

## Figures and Tables

**Table 1:** Baseline characteristics of the study population, analyzed separately according to the LVEF and LVDD quintiles.

	All Patients (N = 630)
Age, years	60.7 (11.8)
Male	527 (83.7%)
BMI, kg/m^2^	24.9 (3.57)
Smoking	358 (56.8%)
Alcohol	262 (41.6%)
Blood pressure	
Systolic, mmHg	119 (17.3)
Diastolic, mmHg	73 (10.7)
NYHA functional class, >II	273 (43.3%)
Comorbidity	
Diabetes	139 (22.1%)
Hypertension	290 (46.0%)
AF	182 (28.9%)
ICM	343 (54.4%)
Stroke	41 (6.51%)
Hyperlipidemia	338 (53.7%)
CRBBB	42 (6.67%)
CLBBB	36 (5.71%)
Primary prevention	145 (23.0%)
Medication	
ACEI/ARB/ARNI	458 (72.7%)
β-blocker	517 (82.1%)
Digoxin	166 (26.3%)
Diuretic	471 (74.8%)
Amiodarone	217 (34.4%)
Biomarker	
NT-proBNP ^a^, pg/ml	936.75 (427.82, 1908.72)
eGFR, ml/min/1.73 m^2^	78.5 (23.6)
Albumin, g/L	41.5 (4.96)

Values are n (%) or mean (standard deviation) where appropriate. ^a^ NT-proBNP was presented as median (IQR). ACEI, angiotensin-converting enzyme inhibitor; AF, atrial fibrillation; ARB, angiotensin II receptor blocker; ARNI, angiotensin receptor-neprilysin inhibitor; BMI, body mass index; CLBBB, complete left bundle branch block; CRBBB, complete right bundle branch block; eGFR, estimated glomerular filtration rate; ICM, ischemic cardiomyopathy; LVEDD, left ventricular end-diastolic diameter; LVEF, left ventricular ejection fraction; NT-proBNP, N-terminal pro-brain natriuretic peptide; NYHA, New York Heart Association.

**Table 2:** Results of multivariable Cox regression for all-cause mortality by LVEF quintiles.

	Model 1		Model 2		Model 3		Model 4	
	HR (95%CI)	*p* Value	HR (95%CI)	*p* Value	HR (95%CI)	*p* Value	HR (95%CI)	*p* Value
LVEF group								
Q1	Ref	-	Ref	-	Ref	-	Ref	-
Q2	0.62 (0.40–0.97)	0.037	0.61 (0.39–0.96)	0.033	0.63 (0.40–0.99)	0.043	0.64 (0.41–1.01)	0.056
Q3	0.65 (0.42–1.02)	0.059	0.63 (0.40–0.99)	0.045	0.66 (0.42–1.03)	0.068	0.72 (0.45–1.15)	0.165
Q4	0.56 (0.34–0.91)	0.020	0.54 (0.33–0.89)	0.016	0.56 (0.34–0.93)	0.024	0.64 (0.38–1.07)	0.086
Q5	0.43 (0.024–0.75)	0.003	0.43 (0.24–0.77)	0.004	0.42 (0.23–0.76)	0.004	0.49 (0.26–0.92)	0.027
P for trend	-	0.003	-	0.003	-	0.004	-	0.030
P for non-linearity	-	0.475	-	0.540	-	0.561	-	0.228
LVEF linear (per 10%)	0.76 (0.64–0.89)	0.001	0.76 (0.64–0.90)	0.002	0.75 (0.62–0.89)	0.001	0.77 (0.64–0.93)	0.006

Model 1 adjusted for age, sex, BMI, systolic blood pressure, NYHA more than II class, the indication of ICD. Model 2 added CRBBB, AF, Diabetes and ICM. Model 3 added use of digoxin, diuretic and ACEI/ARB/ARNI. Model 4 added eGFR, albumin and NT-proBNP. ACEI, angiotensin-converting enzyme inhibitor; AF, atrial fibrillation; ARB, angiotensin II receptor blocker; ARNI, angiotensin receptor-neprilysin inhibitor; BMI, body mass index; CI, confidence interval; CRBBB, complete right bundle branch block; eGFR, estimated glomerular filtration rate; HR, hazard ratio; ICD, implantable cardioverter defibrillator; ICM, ischemic cardiomyopathy; LVEDD, left ventricular end-diastolic diameter; LVEF, left ventricular ejection fraction; NT-proBNP, N-terminal pro-brain natriuretic peptide; NYHA, New York Heart Association.

**Table 3:** Results of multivariable Cox regression for all-cause mortality by LVEDD quintiles.

	Model 1		Model 2		Model 3		Model 4	
	HR (95%CI)	*p* Value	HR (95%CI)	*p* Value	HR (95%CI)	*p* Value	HR (95%CI)	*p* Value
LVEDD group								
Q1	0.37 (0.21–0.63)	<0.001	0.36 (0.20–0.64)	<0.001	0.35 (0.19–0.63)	<0.001	0.39 (0.22–0.70)	0.002
Q2	0.57 (0.35–0.93)	0.024	0.56 (0.34–0.92)	0.023	0.56 (0.34–0.92)	0.022	0.61 (0.36–1.02)	0.058
Q3	0.44 (0.28–0.69)	<0.001	0.40 (0.25–0.64)	<0.001	0.40 (0.25–0.63)	<0.001	0.41 (0.26–0.66)	<0.001
Q4	0.59 (0.38–0.91)	0.018	0.55 (0.36–0.86)	<0.001	0.54 (0.35–9.85)	0.008	0.54 (0.35–0.85)	0.008
Q5	Ref	-	Ref	-	Ref	-	Ref	-
P for trend	-	<0.001	-	<0.001	-	<0.001	-	<0.001
P for nonlinearity	-	0.663	-	0.696	-	0.689	-	0.466
LVEDD linear (per 10 mm)	1.54 (1.30–1.82)	<0.001	1.56 (1.31–1.87)	<0.001	1.57 (1.31–1.88)	<0.001	1.54 (1.27–1.85)	<0.001

Model 1 adjusted for age, sex, BMI, systolic blood pressure, NYHA more than II class, the indication of ICD. Model 2 added CRBBB, AF, Diabetes and ICM. Model 3 added use of digoxin, diuretic and ACEI/ARB/ARNI. Model 4 added eGFR, albumin and NT-proBNP. ACEI, angiotensin-converting enzyme inhibitor; AF, atrial fibrillation; ARB, angiotensin II receptor blocker; ARNI, angiotensin receptor-neprilysin inhibitor; BMI, body mass index; CI, confidence interval; CRBBB, complete right bundle branch block; eGFR, estimated glomerular filtration rate; HR, hazard ratio; ICD, implantable cardioverter defibrillator; ICM, ischemic cardiomyopathy; LVEDD, left ventricular end-diastolic diameter; LVEF, left ventricular ejection fraction; NT-proBNP, N-terminal pro-brain natriuretic peptide; NYHA, New York Heart Association.

**Table 4:** Results of competing risk analysis for free from appropriate shock by LVEF quintiles.

	Model 1		Model 2		Model 3		Model 4	
	HR (95%CI)	*p* Value	HR (95%CI)	*p* Value	HR (95%CI)	*p* Value	HR (95%CI)	*p* Value
LVEF group								
Q1	Ref	-	Ref	-	Ref	-	Ref	-
Q2	1.50 (0.95–2.39)	0.084	1.62 (1.00–2.60)	0.049	1.55 (0.95–2.53)	0.082	1.59 (0.97–2.60)	0.064
Q3	1.04 (0.63–1.72)	0.878	1.19 (0.70–2.03)	0.518	1.16 (0.69–1.96)	0.567	1.21 (0.72–2.04)	0.468
Q4	1.20 (0.72–1.98)	0.484	1.33 (0.79–2.25)	0.287	1.24 (0.73–2.10)	0.425	1.31 (0.77–2.20)	0.320
Q5	0.73 (0.41–1.27)	0.263	0.81 (0.45–1.47)	0.494	0.72 (0.40–1.32)	0.289	0.77 (0.42–1.41)	0.393
P for trend	-	0.475	-	0.796	-	0.590	-	0.761
P for non-linearity	-	0.233	-	0.161	-	0.165	-	0.148

Model 1 adjusted for age, sex, smoking, alcohol, BMI, systolic blood pressure, NYHA more than II class, the indication of ICD. Model 2 added CRBBB, AF, Diabetes and ICM. Model 3 added use of digoxin, diuretic and ACEI/ARB/ARNI. Model 4 added eGFR, albumin and NT-proBNP. ACEI, angiotensin-converting enzyme inhibitor; AF, atrial fibrillation; ARB, angiotensin II receptor blocker; ARNI, angiotensin receptor-neprilysin inhibitor; BMI, body mass index; CI, confidence interval; CRBBB, complete right bundle branch block; eGFR, estimated glomerular filtration rate; HR, hazard ratio; ICD, implantable cardioverter defibrillator; ICM, ischemic cardiomyopathy; LVEDD, left ventricular end-diastolic diameter; LVEF, left ventricular ejection fraction; NT-proBNP, N-terminal pro-brain natriuretic peptide; NYHA, New York Heart Association.

**Table 5:** Results of competing risk analysis for free from appropriate shock by LVEDD quintiles.

	Model 1		Model 2		Model 3		Model 4	
	HR (95%CI)	*p* Value	HR (95%CI)	*p* Value	HR (95%CI)	*p* Value	HR (95%CI)	*p* Value
LVEDD group								
Q1	0.51 (0.29–0.90)	0.021	0.59 (0.33–1.06)	0.078	0.54 (0.30–0.96)	0.037	0.55 (0.31–0.99)	0.048
Q2	0.49 (0.28–0.85)	0.011	0.50 (0.29–0.89)	0.017	0.46 (0.26–0.82)	0.008	0.47 (0.26–0.85)	0.013
Q3	0.78 (0.51–1.20)	0.265	0.84 (0.54–1.30)	0.437	0.81 (0.53–1.24)	0.335	0.83 (0.53–1.29)	0.405
Q4	0.81 (0.53–1.25)	0.341	0.83 (0.54–1.29)	0.409	0.77 (0.50–1.24)	0.248	0.78 (0.50–1.20)	0.260
Q5	Ref	-	Ref	-	Ref	-	Ref	-
P for trend	-	0.004	-	0.019	-	0.008	-	0.014
P for non-linearity	-	0.325	-	0.424	-	0.464	-	0.446
LVEDD linear (per 10 mm)	1.28 (1.09–1.50)	0.002	1.24 (1.04–1.46)	0.012	1.29 (1.08–1.53)	0.004	1.27 (1.06–1.52)	0.008

Model 1 adjusted for age, sex, smoking, alcohol, BMI, systolic blood pressure, NYHA more than II class, the indication of ICD. Model 2 added CRBBB, AF, Diabetes and ICM. Model 3 added use of digoxin, diuretic and ACEI/ARB/ARNI. Model 4 added eGFR, albumin and NT-proBNP. ACEI, angiotensin-converting enzyme inhibitor; AF, atrial fibrillation; ARB, angiotensin II receptor blocker; ARNI, angiotensin receptor-neprilysin inhibitor; BMI, body mass index; CI, confidence interval; CRBBB, complete right bundle branch block; eGFR, estimated glomerular filtration rate; HR, hazard ratio; ICD, implantable cardioverter defibrillator; ICM, ischemic cardiomyopathy; LVEDD, left ventricular end-diastolic diameter; LVEF, left ventricular ejection fraction; NT-proBNP, N-terminal pro-brain natriuretic peptide; NYHA, New York Heart Association.

## Data Availability

The data of this study are available on reasonable request from the corresponding author.

## Supplementary Materials

The following supporting information can be downloaded at: https://www.mdpi.com/article/10.3390/jcdd9120421/s1, Table S1: Baseline characteristics of the study population according to the LVEF and LVDD quintiles; Table S2: Univariable Cox regression for the prediction of all-cause mortality and appropriate shock; Table S3: Multivariable regression results according to echo parameter quintiles and clinical outcomes in model 4; Table S4: Statistical association of baseline variables with all-cause mortality and appropriate shock; Table S5: Incremental prognostic value of LVEF and LVEDD for clinical outcomes; Table S6: Interaction analysis for prediction of outcomes; Table S7: Prediction of all-cause mortality and appropriate shock using complete-case (N = 535).

## Nonstandard Abbreviations and Acronyms

ACEI	angiotensin-converting enzyme inhibitor
AF	atrial fibrillation
ARB	angiotensin II receptor blocker
ARNI	angiotensin receptor-neprilysin inhibitor
BMI	body mass index
CI	confidence interval
CLBBB	complete left bundle branch block
CRBBB	complete right bundle branch block
eGFR	estimated glomerular filtration rate
HR	hazard ratio
ICD	implantable cardioverter defibrillator
ICM	ischemic cardiomyopathy
IDI	integrated discrimination index
LVEDD	left ventricular end-diastolic diameter
LVEF	left ventricular ejection fraction
NICM	non-ischemic cardiomyopathy
NRI	net reclassification index
NT-proBNP	N-terminal pro-brain natriuretic peptide
NYHA	New York Heart Association
SCD	sudden cardiac death
