# Supplementary material for: Prognostic Implications of Left Ventricular Ejection Fraction and Left Ventricular End-Diastolic Diameter on Clinical Outcomes in Patients with ICD
Source: J Cardiovasc Dev Dis. 2022 Nov 28;9(12):421. doi: 10.3390/jcdd9120421 (PMC9782887; doi:10.3390/jcdd9120421)
Supplement: Supplementary file 1 [file jcdd-09-00421-s001.zip › jcdd-1962713-supplementary.pdf]

SUPPLEMENTAL MATERIAL

Prognostic Implications of Left Ventricular Ejection Fraction and Left Ventricular End-Diastolic Diameter on Clinical Outcomes in Patients with ICD

Authors: Sijing Cheng, MD*; Yu Deng, MD*; Hao Huang, MD; Xi Liu, MD; Yu Yu, MD; Xuhua Chen, MD; Min Gu, MD; Hongxia Niu, MD; Wei Hua, MD, PhD, FHRS

* contributed equally

Affiliation: The Cardiac Arrhythmia Center, State Key Laboratory of Cardiovascular Disease, Fuwai Hospital, National Center for Cardiovascular Diseases, Chinese Academy of Medical Sciences and Peking Union Medical College, No.167 North Lishi Road, Beijing 100037, China

Correspondence:

Wei Hua, PhD

State Key Laboratory of Cardiovascular Disease, Arrhythmia Center, Fuwai Hospital, National Center for Cardiovascular Diseases, Chinese Academy of Medical Sciences and Peking Union Medical College, No.167 North Lishi Road, Beijing 100037, China

E-mail: drhuaweifw@sina.com

Table S1. Baseline characteristics of the study population according to the LVEF and LVDD quintiles

	LVEF						LVEDD					
	Q1<br><27%<br>N=126	Q2<br>27-33%<br>N=122	Q3<br>33-38%<br>N=125	Q4<br>38-48%<br>N=128	Q5<br>≥48%<br>N=129	P value	Q1<br><57mm<br>N=121	Q2<br>57-61mm<br>N=109	Q3<br>61-66mm<br>N=141	Q4<br>66-72mm<br>N=131	Q5<br>≥72mm<br>N=128	P value
Age, years	57.3(12.4)	59.8(12.0)	62.5(10.7)	62.0(12.1)	61.8(11.3)	0.002*	63.4(11.4)	61.6(12.7)	60.7(11.1)	60.5(11.8)	57.6(11.8)	0.003*
Male	101(80.2%)	103(84.4%)	109(87.2%)	107(83.6%)	107(82.9%)	0.667	90(74.4%)	87(79.8%)	120(85.1%)	118(90.1%)	112(87.5%)	0.006*
BMI, kg/m^2	24.4(3.82)	25.2(3.86)	24.8(3.10)	25.0(3.26)	25.0(3.74)	0.391	24.3(3.82)	24.9(3.46)	24.8(3.14)	25.0(3.33)	25.5(4.02)	0.149
Smoking	69(54.8%)	66(54.1%)	70(56.0%)	69(53.9%)	84(65.1%)	0.322	70(57.9%)	60(55.0%)	73(51.8%)	84(64.1%)	71(55.5%)	0.331
Alcohol	55(43.7%)	51(41.8%)	47(37.6%)	49(38.3%)	60(46.5%)	0.574	41(33.9%)	52(47.7%)	50(35.5%)	66(50.4%)	53(41.4%)	0.027*
Blood pressure												
Systolic, mmHg	114(15.6)	115(18.5)	118(14.0)	122(16.4)	127(18.5)	<0.001*	124(16.9)	123(17.8)	118(17.3)	117(15.1)	115(17.8)	<0.001*
Diastolic, mmHg	71.4(10.2)	74.0(11.9)	72.3(9.6)	74.4(9.9)	74.8(11.5)	0.051	74.9(11.4)	74.3(9.5)	73.1(10.6)	72.6(10.5)	72.4(11.1)	0.272
NYHA functional class, >II	79(62.7%)	66(54.1%)	59(47.2%)	48(37.5%)	21(16.3%)	<0.001*	29(24.0%)	38(34.9%)	60(42.6%)	67(51.1%)	79(61.7%)	<0.001*
Comorbidity												
Diabetes	29(23.0%)	32(26.2%)	25(20.0%)	25(19.5%)	28(21.7%)	0.718	23(19.0%)	25(22.9%)	35(24.8%)	27(20.6%)	29(22.7%)	0.824
Hypertension	41(32.5%)	50(41.0%)	64(51.2%)	63(49.2%)	72(55.8%)	0.002*	81(66.9%)	58(53.2%)	79(56.0%)	69(52.7%)	51(39.8%)	0.001*
AF	42(33.3%)	42(34.4%)	39(31.2%)	28(21.9%)	31(24.0%)	0.095	27(22.3%)	34(31.2%)	40(28.4%)	43(32.8%)	38(29.7%)	0.423
ICM	39(31.0%)	55(45.1%)	75(60.0%)	78(60.9%)	96(74.4%)	<0.001*	93(76.9%)	67(61.5%)	81(57.4%)	63(48.1%)	39(30.5%)	<0.001*
Stroke	9(7.1%)	10(8.2%)	10(8.0%)	8(6.3%)	4(3.1%)	0.465	5(4.13%)	5(4.59%)	15(10.6%)	10(7.63%)	6(4.69%)	0.152
Hyperlipidemia	47(37.3%)	66(54.1%)	71(56.8%)	71(55.5%)	83(64.3%)	<0.001*	81(66.9%)	58(53.2%)	79(56.0%)	69(52.7%)	51(39.8%)	0.001*
CRBBB	9(7.1%)	7(5.7%)	9(7.2%)	11(8.6%)	6(4.7%)	0.758	7(5.79%)	3(2.75%)	13(9.22%)	12(9.16%)	7(5.47%)	0.205
CLBBB	11(8.7%)	7(5.7%)	9(7.2%)	11(8.6%)	6(4.7%)	0.585	3(2.48%)	3(2.75%)	5(3.55%)	9(6.87%)	16(12.5%)	0.002**

Primary prevention	54(42.9%)	59(48.4%)	32(25.6%)	0	0	<0.001*	3(2.48%)	13(11.9%)	35(24.8%)	40(30.5%)	54(42.2%)	<0.001*
Medication												
ACEI/ARB/ARNI	92(73.0%)	90(73.8%)	94(75.2%)	99(77.3%)	83(64.3%)	0.171	81(66.9%)	83(76.1%)	107(75.9%)	97(74.0%)	90(0.3%)	0.422
β-blocker	97(77.0%)	98(80.3%)	101(80.8%)	113(88.3%)	108(83.7%)	0.185	103(85.1%)	95(87.2%)	117(83.0%)	107(81.7%)	95(74.2%)	0.087
Digoxin	54(42.9%)	43(35.2%)	35(28.0%)	21(16.4%)	13(10.1%)	<0.001*	12(9.92%)	22(20.2%)	40(28.4%)	40(30.5%0	52(40.6%)	<0.001*
Diuretic	115(91.3%)	105(86.1%)	104(83.2%)	92(71.9%)	55(42.6%)	<0.001*	58(47.9%)	77(70.6%)	109(77.3%)	109(83.2%)	118(92.2%)	<0.001*
Amiodarone	37(29.4%)	25(20.5%)	39(31.2%)	56(43.8%)	60(46.5%)	<0.001*	54(44.6%)	42(38.5%)	45(31.9%)	44(33.6%)	32(25.0%)	0.018*
Biomarker												
NT-proBNP[a], pg/ml	1591(811,3795)	1286(705,2475)	942(428,1796)	708(331,1626)	473(233,880)	<0.001*	578(250,1274)	697(308,1384)	970(615,1816)	1075(502,2228)	1429(663,2778)	<0.001*
eGFR, ml/min/1.73m^2	79.9(20.6)	77.6(24.0)	74.4(24.8)	76.5(25.0)	83.7(22.6)	0.021*	82.8(24.9)	80.5(23.8)	76.2(22.3)	74.9(22.7)	78.7(24.0)	0.057
Albumin, g/L	41.9(4.60)	41.8(4.60)	41.4(5.38)	41.6(5.40)	40.8(4.76)	0.390	41.5(4.82)	41.2(5.38)	41.2(5.52)	41.7(4.50)	42.0(4.54)	0.639

Values are n (%) or mean (standard deviation) where appropriate.

* denotes P<0.05.

[a]NT-proBNP was presented as median (IQR).

AF, atrial fibrillation; ARNI, angiotensin receptor-neprilysin inhibitor; BMI, body mass index; CLBBB, complete left bundle branch block; CRBBB, complete right bundle branch block; eGFR, estimated glomerular filtration rate; ICM, ischemic cardiomyopathy; LVEDD, left ventricular end-diastolic diameter; LVEF, left ventricular ejection fraction.

Table S2. Univariable Cox regression for the prediction of all-cause mortality and appropriate shock.

	All-cause mortality		Shock	
	HR (95%CI)	P value	HR (95%CI)	P value
Age, years	1.01(0.99-1.02)	0.23	0.99(0.97-1)	0.026*
Female	1.11(0.75-1.64)	0.60	0.57(0.36-0.92)	0.021*
BMI, kg/m^2	0.97(0.92-1.01)	0.97	0.98(0.94-1.03)	0.415
Alcohol	0.85(0.62-1.16)	0.31	1.57(1.16-2.12)	0.003*
Smoking	0.94(0.70-1.28)	0.71	1.40(1.03-1.91)	0.033*
Systolic BP, mmHg	0.98(0.97-0.99)	<0.001*	0.99(0.98-1.00)	0.069
NYHA functional class, >II	2.25(1.65-3.07)	<0.001*	0.99(0.73-1.34)	0.95
NSVT	0.97(0.62-1.52)	0.88	1.16(0.77-1.75)	0.48
Primary prevention	1.30(0.91-1.85)	0.16	0.89(0.61-1.32)	0.57
ICM	0.83(0.61-1.12)	0.22	0.57(0.42-0.77)	<0.001*
Diabetes	1.4(0.98-2)	0.061	0.83(0.57-1.22)	0.352
AF	1.3(0.94-1.79)	0.12	1.13(0.82-1.56)	0.46
Hyperlipidemia	0.89(0.66-1.21)	0.46	0.72(0.53-0.97)	0.031*
Stroke	0.94(0.48-1.85)	0.87	1.00(0.50-1.97)	0.99
CRBBB	2.24(1.42-3.55)	0.001*	0.48(0.22-1.03)	0.06
Amiodarone	1(0.73-1.36)	0.996	0.86(0.63-1.17)	0.33
ACEI/ARB/ARNI	0.57(0.42-0.78)	<0.001*	0.98(0.7-1.36)	0.90
β-blocker	0.82(0.58-1.17)	0.28	1.26(0.87-1.82)	0.22
Digoxin	1.52(1.11-2.08)	0.01*	0.79(0.55-1.12)	0.18
Diuretic	1.46(0.99-2.13)	0.053	1.03(0.73-1.45)	0.87
NT-proBNP[a], pg/ml	4.26(3.00-6.06)	<0.001*	1.01(0.73-1.40)	0.94

eGFR, ml/min/1.73m^2	0.99(0.98-1)	0.005*	1.00(0.99-1.01)	0.74
Albumin, g/L	0.96(0.94-0.99)	0.01*	1.00(0.96-1.03)	0.91
LVEF quintiles				
Q1	Ref	-	Ref	-
Q2	0.59(0.38-0.92)	0.02*	1.35(0.74-2.13)	0.192
Q3	0.65(0.42-0.99)	0.046*	0.97(0.60-1.57)	0.904
Q4	0.47(0.30-0.75)	0.001*	1.15(0.73-1.82)	0.539
Q5	0.30(0.18-0.51)	<0.001*	0.74(0.44-1.23)	0.245
LVEDD quintiles				
Q1	0.32(0.19-0.53)	<0.001*	0.53(0.31-0.88)	0.015*
Q2	0.49(0.31-0.79)	0.003*	0.53(0.32-0.88)	0.015*
Q3	0.41(0.26-0.64)	<0.001*	0.78(0.52-1.17)	0.238
Q4	0.61(0.40-0.93)	0.02*	0.86(0.57-1.30)	0.479
Q5	Ref	-	Ref	-
LVEF, %	0.96(0.95-0.98)	<0.001*	0.99(0.98-1.00)	0.17
LVEDD, mm	1.05(1.03-1.06)	<0.001*	1.02(1.00-1.04)	0.006*

* denotes P<0.05.

[a]NT-proBNP was included as a continuous log_{10}-transformed value.

AF, atrial fibrillation; ARNI, angiotensin receptor-neprilysin inhibitor; BMI, body mass index; CRBBB, complete right bundle branch block; eGFR, estimated glomerular filtration rate; ICM, ischemic cardiomyopathy; LVEDD, left ventricular end-diastolic diameter; LVEF, left ventricular ejection fraction.

Table S3 multivariable regression results according to echo parameter quintiles and clinical outcomes in model 4

	All-cause mortality		Shock	
	LVEF	LVEDD	LVEF	LVEDD
Age, years	1.00(0.99-1.02)	1.01(0.99-1.02)	0.99(0.98-1.01)	0.99(0.98-1.01)
Female	0.91(0.60-1.39)	0.88(0.56-1.37)	0.66(0.38-1.14)	0.69(0.40-1.19)
Smoking	-	-	1.39(0.97-2.01)	1.25(0.87-1.81)
Alcohol	-	-	1.28(0.90-1.81)	1.34(0.94-1.91)
BMI, kg/m^2	1.01(0.96-1.06)	1.00(0.95-1.05)	0.97(0.93-1.01)	0.97(0.92-1.01)
Systolic BP, mmHg	0.99(0.98-1.00)	0.99(0.98-1.00)	1.00(0.99-1.01)	1.00(0.99-1.00)
NYHA functional class, > II	1.66(1.18-2.33)	1.65(1.17-2.32)	0.99(0.70-1.41)	0.96(0.68-1.36)
Primary prevention	0.80(0.86-1.33)	0.87(0.59-1.29)	0.76(0.48-1.20)	0.73(0.48-1.12)
CRBBB	2.13(1.31-3.47)	2.32(1.42-3.79)	0.60(0.29-1.27)	0.58(0.28-1.22)
AF	1.01(0.70-1.45)	1.03(0.72-1.47)	1.04(0.75-1.45)	1.13(0.82-1.57)
Diabetes	1.43(0.99-2.07)	1.40(0.97-2.03)	0.91(0.61-1.34)	0.89(0.61-1.31)
ICM	0.92(0.65-1.31)	0.98(0.69-1.40)	0.52(0.36-0.74)	0.56(0.39-0.78)
Digoxin	1.12(0.78-1.59)	1.12(0.79-1.60)	0.66(0.44-0.99)	0.62(0.41-0.92)
Diuretic	1.16(0.56-1.32)	0.88(0.57-1.35)	0.95(0.65-1.38)	0.97(0.68-1.39)
ACEI/ARB/ARNI	0.70(0.50-0.99)	0.70(0.50-0.99)	0.97(0.68-1.38)	1.02(0.72-1.43)
eGFR, $ml/min/1.73m^2$	0.99(0.99-1.00)	0.99(0.99-1.00)	1.00(0.99-1.00)	1.00(0.99-1.01)
Albumin	0.97(0.94-1.00)	0.97(0.94-0.99)	0.99(0.96-1.03)	1.00(0.97-1.04)
NT-proBNP, pg/ml	1.00(1.00-1.00)	1.00(1.00-1.00)	1.00(1.00-1.00)	1.00(1.00-1.00)

AF, atrial fibrillation; ARNI, angiotensin receptor-neprilysin inhibitor; BMI, body mass index; CRBBB, complete right bundle branch block; eGFR, estimated glomerular filtration rate; ICM, ischemic cardiomyopathy; LVEDD, left ventricular end-diastolic diameter; LVEF, left ventricular ejection fraction.

Table S4. Statistical association of baseline variables with all-cause mortality and appropriate shock

	All-cause mortality			Shock		
	Survivors (N=462)	Non-survivors (N=168)	P value	Treat (N=156)	Non-treat (N=474)	P value
Age, years	60.3(11.4)	61.7(12.9)	0.21	59.2(12.2)	61.2(11.7)	0.08
Male	390(84.4%)	137(81.5%)	0.46	138(88.5%)	389(82.1%)	0.08
BMI, kg/m^2	25.1(3.52)	24.4(3.67)	0.028*	24.6(3.68)	25.0(3.53)	0.196
Blood pressure						
Systolic, mmHg	121(16.2)	115(19.3)	<0.001*	117(15.0)	120(17.9)	0.021*
Diastolic, mmHg	74(10.3)	72(11.6)	0.029*	74(9.58)	73(11.0)	0.684
NYHA functional class, >II	174(37.7%)	99(58.9%)	<0.001*	70(44.9%)	203(43.8%)	0.72
LVEF, %	38.6(11.9)	33.7(10.2)	<0.001*	36.1(10.2)	37.7(12.1)	0.12
LVEDD, mm	63.1(9.4)	67.3(10.0)	<0.001*	66.1(9.2)	63.6(9.8)	0.004*
Comorbidity						
Diabetes	98(21.2%)	41(24.4%)	0.46	28(17.9%)	111(23.4%)	0.19
Hypertension	216(46.8%)	74(44.0%)	0.61	63(40.4%)	227(47.9%)	0.124
AF	128(27.7%)	54(32.1%)	0.324	50(32.1%)	132(27.8%)	0.37
ICM	258(55.8%)	85(50.6%)	0.28	65(41.7%)	278(58.6%)	<0.001*
Stroke	32(6.93%)	9(5.36%)	0.601	8(5.13%)	33(6.96%)	0.54
Hyperlipidemia	256(55.4%)	82(48.8%)	0.17	70(44.9%)	268(56.5%)	0.015*
CRBBB	21(4.55%)	21(12.5%)	0.001*	6(3.85%)	36(7.59%)	0.149
CLBBB	28(6.06%)	8(4.76%)	0.67	8(5.13%)	28(5.91%)	0.87
Primary prevention	105(22.7%)	40(23.8%)	0.86	30(19.2%)	115(24.324.3%)	0.236
Medication						

ACEI/ARB/ARNI	353(76.4%)	105(62.5%)	0.001*	111(71.2%)	347(73.2%)	0.69
Beta-blocker	390(84.4%)	127(75.6%)	0.015*	127(81.4%)	390(82.3%)	0.90
Digoxin	105(22.7%)	61(36.3%)	0.001*	37(23.7%)	129(27.2%)	0.45
Diuretic	336(72.7%)	135(80.4%)	0.065	117(75.0%)	354(74.7%)	1.0
Amiodarone	151(32.7%)	66(39.3%)	0.15	57(35.5%)	160(33.8%)	0.59
NT-proBNP[a], pg/ml	727(345,1456)	1840(939;3240)	<0.001*	1014(471;2018)	876(417;1887)	0.24
eGFR_MDRD, ml/min/1.73m^2	79.4(22.3)	75.9(26.8)	0.13	80.1(24.8)	77.9(23.2)	0.34
Albumin, g/L	41.8(5.16)	40.8(4.30)	0.016*	41.6(5.13)	41.5(4.91)	0.84

* denotes P<0.05.

[a]NT-proBNP was presented as median (IQR).

AF, atrial fibrillation; ARNI, angiotensin receptor-neprilysin inhibitor; BMI, body mass index; CLBBB, complete left bundle branch block; CRBBB, complete right bundle branch block; eGFR, estimated glomerular filtration rate; ICM, ischemic cardiomyopathy; LVEDD, left ventricular end-diastolic diameter; LVEF, left ventricular ejection fraction.

Table S5. Incremental prognostic value of LVEF and LVEDD for clinical outcomes.

	Model 1					Model 2				
	Clinical	Clinical + LVEF	P value	Clinical + LVEDD	P value	Clinical	Clinical + LVEF	P value	Clinical + LVEDD	P value
**All-cause mortality**										
C-statistic	0.65(0.60,0.70)	0.676(0.63,0.72)	-	0.68(0.63,0.73)	-	0.67(0.62,0.72)	0.69(0.64,0.73)	-	0.69(0.65,0.74)	-
IDI	-	0.017(0.003,0.040)	0.013	0.038(0.013,0.070)	<0.001	-	0.017(0.004,0.040)	0.02	0.040(0.013-0.075)	<0.0001
NRI (95%CI)	-	0.185(0.053-0.306)	-	0.277(0.140-0.382)	-	-	0.124(0.006,0.249)	-	0.235(0.105,0.352)	-
Likelihood-ratio test, Δχ2	-	11.169	0.0008	23.508	<0.0001	-	10.449	0.001	23.212	<0.0001
**Shock**										
C-statistic	0.61(0.57,0.66)	0.618(0.57,0.66)	-	0.625(0.58-0.67)	-	0.65(0.60,0.69)	0.65(0.60,0.69)	-	0.65(0.60,0.69)	-
IDI	-	0.011(-0.002,0.037)	0.14	0.018(-0.003,0.055)	0.133	-	0.009(-0.002,0.032)	0.179	0.015(-0.005,0.049)	0.186
NRI (95%CI)	-	0.08(-0.055,0.223)	-	0.076(0,0.227)	-	-	0.09(-0.066,0.188)	-	0.064(-0.01,0.193)	-
Likelihood-ratio test, Δχ2	-	1.311	0.25	7.225	0.007	-	0.429	0.51	4.97	0.026
	Model 3					Model 4				
	Clinical	Clinical + LVEF	P value	Clinical + LVEDD	P value	Clinical	Clinical + LVEF	P value	Clinical + LVEDD	P value
**All-cause mortality**										
C-statistic	0.68(0.63,0.73)	0.70(0.66,0.74)	-	0.70(0.66,0.75)	-	0.70(0.65,0.74)	0.71(0.67,0.76)	-	0.71(0.67,0.76)	-
IDI (95%CI)	-	0.017(0.003,0.047)	0.020	0.041(0.014,0.072)	<0.0001	-	0.015(0.002,0.038)	0.007	0.037(0.011,0.069)	<0.0001
NRI (95%CI)	-	0.100(-0.021,0.236)	-	0.244(0.112,0.352)	-	-	0.129(0.007,0.248)	-	0.243(0.091,0.320)	-
Likelihood-ratio test, Δχ2	-	10.606	0.001	23.129	<0.0001	-	7.779	0.005	19.574	<0.0001
**Shock**										
C-statistic	0.66(0.61,0.70)	0.66(0.62,0.70)	-	0.66(0.61,0.70)	-	0.66(0.62,0.70)	0.66(0.62,0.71)	-	0.66(0.62,0.71)	-
IDI (95%CI)	-	0.006(-0.007,0.028)	0.319	0.014(-0.005,0.052)	0.186	-	0.005(-0.006,0.027)	0.412	0.013(-0.006,0.044)	0.140

NRI (95%CI)	-	0.095(-0.045,0.185)	-	0.103(-0.002,0.221)	-	-	0.102(-0.053,0.185)	-	0.136(-0.006,0.202)	-
Likelihood-ratio test, $\Delta\chi 2$	-	0.941	0.33	6.603	0.01	-	0.597	0.44	5.814	0.02

CI, confidence interval; IDI, integrated discrimination index; LVEDD, left ventricular end-diastolic diameter; LVEF, left ventricular ejection fraction; NRI, net reclassification index.

Table S6. Interaction analysis for prediction of outcomes.

	LVEF		LVEDD	
	All-cause death	Appropriate shock	All-cause death	Appropriate shock
Indication	0.21	0.77	0.51	0.28
ICM	0.09	0.99	0.34	0.79
Sex	0.36	0.56	0.48	0.21
Diabetes	0.60	0.36	0.59	0.95
Hyperlipidemia	0.10	0.06	0.07	0.42
Digoxin	0.39	0.09	0.58	0.11
Diuretic	0.47	0.52	0.47	0.85
Amiodarone	0.38	0.43	0.88	0.30

ICM, ischemic cardiomyopathy; LVEDD, left ventricular end-diastolic diameter; LVEF, left ventricular ejection fraction.

Table S7. Prediction of all-cause mortality and appropriate shock using complete-case (N=535).

	Model 4	
	HR (95%CI)	P value
All-cause mortality		
LVEF linear (per 10%)	0.78(0.63,0.96)	0.020
LVEDD linear (per 10mm)	1.51(1.23,1.86)	<0.001
Appropriate shock		
LVEF trend	0.92(0.81,1.05)	0.230
LVEDD (per 10mm)	1.25(1.04,1.51)	0.017

Model 4 for all-cause mortality adjusted for age, sex, BMI, systolic blood pressure, NYHA more than II class, the indication of ICD, CRBBB, AF, Diabetes, ICM, use of digoxin, diuretic and ACEI/ARB/ARNI, eGFR, albumin and NT-proBNP. Model 4 for appropriate shock further adjusted for smoking and alcohol.

AF, atrial fibrillation; ARNI, angiotensin receptor-neprilysin inhibitor; BMI, body mass index; CRBBB, complete right bundle branch block; eGFR, estimated glomerular filtration rate; HR, hazard ratio; ICD, implantable cardioverter defibrillator; ICM, ischemic cardiomyopathy; LVEDD, left ventricular end-diastolic diameter; LVEF, left ventricular ejection fraction.
